# Influence of Neighboring Orientations on Oriented Stability in Grain-Boundary Regions of Non-Oriented Silicon Steel

**DOI:** 10.3390/ma19173733

**Published:** 2026-09-02

**Authors:** Xi Chen, Guojin Zhang, Fang Zhang, Yuhui Sha

**Affiliations:** 1 School of Mechanical Engineering and Automation, Dalian Polytechnic University, Dalian 116034, China; 2 Key Laboratory for Anisotropy and Texture of Materials (Ministry of Education), Northeastern University, Shenyang 110819, China

**Keywords:** oriented stability, grain-boundary region, non-oriented silicon steel, neighboring orientations, crystal plasticity simulation

## Abstract

Orientation rotation in grain-boundary regions plays a critical role in controlling the crystallographic texture of metallic materials. In this study, the ideal λ texture ({001}<uv0>) in non-oriented silicon steel is chosen as the target orientation. The oriented stability in grain-boundary regions during cold rolling is systematically investigated by combining crystal plasticity simulations and quasi in situ electron backscatter diffraction (EBSD) experiments. Oriented stability is defined as the rate of change in the misorientation angle between an arbitrary orientation and the target orientation, thereby quantifying the rotational tendency relative to the target in grain-boundary regions. The results reveal that the oriented stability in grain-boundary regions is highly sensitive to both the initial and neighboring orientations. For initial orientations near the critical boundary separating convergence and divergence zones, the oriented stability is highly susceptible to neighboring orientations, with some neighboring orientations even reversing the rotation direction. In contrast, when the initial orientation is far from this critical boundary, the influence of neighboring orientations becomes weaker. Furthermore, the concept of contributed oriented stability is introduced to statistically evaluate the effect of different neighboring texture components in polycrystals. This work elucidates the underlying mechanism of orientation rotation in grain-boundary regions, and provides a new theoretical framework and a quantitative strategy for optimizing favorable textures.

## 1. Introduction

Crystallographic texture is a ubiquitous feature of metallic materials and exerts a profound influence on their mechanical and physical properties. Both orientation rotation during plastic deformation and recrystallization upon annealing can substantially alter the texture. In particular, grain-boundary regions experience additional constraints imposed by neighboring grains during deformation, leading to rotation behaviors that differ markedly from those in grain interiors [1,2]. Moreover, because grain boundaries serve as preferential nucleation sites for recrystallization [3,4], the orientation evolution in these regions is crucial for effective texture control.

Non-oriented silicon steel is an important soft magnetic material widely used in high-precision applications such as traction motors for new energy vehicles, robotics, and low-altitude aircraft [5,6]. In this material, the λ texture (<001>//ND, normal direction) is beneficial to magnetic properties [7,8,9]; however, it is difficult to strengthen through plastic deformation. Consequently, enhancing favorable texture components by manipulating orientation rotation in grain-boundary regions has become a key research priority, not only for non-oriented silicon steel but also for texture-controlled metallic materials in general.

During plastic deformation, different initial orientations rotate along distinct paths and tend to converge toward orientations of high stability [10,11]. Orientation stability thus serves as a fundamental descriptor for interpreting texture evolution. According to the classical criterion proposed by Clement [12], a stable orientation exhibits zero rotation rate and is an orientation toward which all neighboring orientations in Euler space converge. To relax the stringency of this criterion, Tóth et al. [13,14,15] and Li et al. [16,17,18] developed a series of stability parameters and identified ideal orientations with high stability under various deformation modes. Orientation stability describes the tendency of an initial orientation to deviate from itself and identifies stable end orientations under a given deformation mode. In practice, however, the target orientation that is favorable for material properties may be stable, metastable, or even unstable. In addition, other orientations can exhibit varying tendencies to approach or deviate from that target orientation. Therefore, evaluating and controlling the rotation behavior of an initial orientation relative to a designated target orientation constitutes a central challenge in texture optimization. To address this, the concept of oriented stability was introduced [19]; it precisely quantifies the rotational tendency of an initial orientation relative to a target orientation through the rate of change in their misorientation angle.

During plastic deformation, a grain can be approximately partitioned into the grain interior and the grain-boundary region. Whereas the grain interior rotates relatively freely, lattice rotation in the grain-boundary region is restricted by neighboring grains, resulting in lattice curvature. These curvatures and the associated strain gradients are accommodated by geometrically necessary dislocations (GNDs) [20,21]. GNDs contribute to forest hardening, and their spatially heterogeneous distribution generates back stresses that promote the formation of orientation gradients in grain-boundary regions [22,23]. The orientation evolution in these regions is sensitive to both the initial orientation and the neighboring orientations. Tsuji et al. [24] examined cold-rolled ferritic stainless steel and observed that in grains with {001}⟨510⟩–⟨320⟩ orientations, deformation bands tend to appear near grain boundaries, whereas {001}⟨110⟩-oriented grains preserve a homogeneous orientation throughout. Chen et al. [25] noted that scattered {001}⟨uv0⟩ orientations, when affected by neighboring grains, exhibit a stronger rotation at grain boundaries compared to the grain interior. Nagarajan et al. [26] put forward that a grain preferentially rotates toward its neighboring grain that gives the smallest misorientation angle. Zaefferer et al. [27] and Mishra et al. [28] further demonstrated that the rotation in grain-boundary regions correlates with the disorientation between neighboring grains.

Electron backscatter diffraction (EBSD) experiments and crystal plasticity simulations have been extensively employed to investigate orientation rotation in grain-boundary regions. EBSD-derived parameters—including kernel average misorientation (KAM), grain reference orientation deviation (GROD) [29,30], gradient average severity (GAS), and boundary effective thickness (BET) [31,32]—effectively characterize the orientation gradient features in these regions. In crystal plasticity modeling, considerable effort has been devoted to incorporating dislocation–grain-boundary interactions into conventional frameworks to accurately simulate orientation rotation near grain boundaries. To reproduce the heterogeneous deformation in grain-boundary regions, Lim et al. [33] incorporated into a crystal plasticity model both the back stress and the resistance that grain boundaries impose on dislocation transmission. In a different approach, Chakraborty et al. [34] proposed a crystal plasticity formulation that concurrently handles the transport of dislocations inside grains and their transfer across grain boundaries, thereby capturing the deformation gradients that develop near boundaries. Zhang et al. [35] introduced a nonlocal crystal plasticity framework in which a flux term describes the spatial redistribution of dislocations driven by their motion. Beyond these continuum-based models, the evolution of GNDs and orientation gradients in grain-boundary regions has also been effectively simulated using strain-gradient viscoplastic self-consistent models [36], elastoplastic self-consistent models [37], and molecular dynamics simulations [38].

Most previous studies have focused on the orientation stability differences between grain interiors and grain-boundary regions, whereas the oriented stability of grain-boundary regions relative to a specific target orientation remains largely unexplored. In particular, the influence of different neighboring orientations on this oriented stability has not yet been systematically reported. In the present work, the ideal λ orientation in non-oriented silicon steel is selected as the target orientation, and the oriented stability of initial scattered λ orientations under various neighboring orientations is systematically investigated in grain-boundary regions. This study aims to establish an approach to optimize favorable textures by controlling orientation rotation in grain-boundary regions.

## 2. Calculation and Experiment Methods

### 2.1. Calculation Model

Oriented stability [19] refers to the variation rate of the misorientation angle between a given orientation and a specific target orientation during deformation. Assuming that the target orientation is $g_{A\;}(\varphi_{1}^{A},\;\Phi^{A},\;\varphi_{2}^{A})$, and an arbitrary orientation is $g_{B}\;(\varphi_{1}^{B},\;\Phi^{B},\;\varphi_{2}^{B})$, the corresponding misorientation angle $\theta_{\mathrm{AB}}$ can be computed via the matrix $M_{B\to A}$:

(1) $$\theta_{\mathrm{AB}}=\arccos\{[\mathrm{tr}(M_{B\to A})\;-\;1]/2\}$$

(2) $$M_{B\to A}=g_{A}g_{B}^{-1}$$

For a strain increment ∆*ε* applied over a time increment ∆*t*, orientation $g_{B}$ evolves to $g_{B^{\prime }}\;({\varphi_{1}^{B}\;+\dot{\;\varphi }}_{1}^{B}∆t,\;{\Phi^{B}\;+\dot{\;\Phi }}^{B}∆t,\;{\varphi_{2}^{B}\;+\;\dot{\varphi }}_{2}^{B}∆t)$. Consequently, the post-deformation misorientation angle can be expressed as

(3) $$\theta_{AB^{\prime }}\;=\arccos\{[\mathrm{tr}(M_{B^{\prime }\to A})\;-\;1]/2\}$$

Thus, oriented stability is expressed as the variation in misorientation divided by (∆ε × ∆t):

(4) $$\dot{\theta }(g)\;=\;(\theta_{AB^{\prime }}\;-\;\theta_{\mathrm{AB}})/(∆\varepsilon \;\times \;∆t)$$

A value of $\dot{\theta }\;<\;0$ indicates that the initial orientation converges toward the target orientation, $\dot{\theta \;}>\;0$ indicates divergence, and $\dot{\theta \;}=\;0$ corresponds to a stable orientation relative to the target.

Rate-dependent crystal plasticity models [19,25] were adopted to calculate the oriented stability. The slip rates $\dot{\gamma }$ from all slip systems collectively contribute to the plastic strain rate $\dot{\varepsilon }$:

(5) $$\dot{\varepsilon }\;=\;\frac{1}{2}\sum_{\alpha }^{k}\left(s^{\alpha }⨂m^{\alpha }\;+\;m^{\alpha }⨂s^{\alpha }\right){\dot{\gamma }}^{\alpha }$$

Here α runs from 1 to k; s^α^ and m^α^ are unit vectors along the slip direction and normal to the slip plane, respectively.

For the calculation of slip rate, a rate-dependent formulation [39] is employed in the grain interior:

(6) $${\dot{\gamma }}^{\alpha }\;={\dot{\;a}}^{\alpha }{\left|\frac{\tau^{\alpha }}{g^{\alpha }}\right|}^{n}\mathrm{sgn}(\tau^{\alpha })$$

Here, the flow rule adopts ${\dot{a}}^{\alpha }$ = 0.001 s^−1^ as the reference strain rate and n = 20 as the rate-sensitivity exponent; the resolved shear stress and strength for system α are denoted by $\tau^{\alpha }$ and $g^{\alpha }$, respectively.

For grain-boundary regions, grain boundaries impede dislocation motion in their vicinity, thus posing an obstacle stress (*τ*_obs_) that serves as supplementary resistance to slip system activation:

(7) $${\dot{\gamma }}^{\alpha }\;={\dot{\;a}}^{\alpha }{\left|\frac{\tau_{\mathrm{eff}}^{\alpha }}{g^{\alpha }}\right|}^{n}\mathrm{sgn}(\tau_{\mathrm{eff}}^{\alpha })$$

(8) $$\tau_{\mathrm{eff}}^{\alpha }=\tau^{\alpha }\;-{\;\tau }_{\mathrm{obs}}^{\alpha }(\left|\tau^{\alpha }\right|\;>\;\tau_{\mathrm{obs}}^{\alpha })$$

(9) $$\tau_{\mathrm{eff}}^{\alpha }=0\;(\left|\tau^{\alpha }\right|\;\leq {\;\tau }_{\mathrm{obs}}^{\alpha })$$

*τ*_obs_ is determined by the slip transmissivity *N* at the grain boundary:

(10) $$\tau_{\mathrm{obs}}\;=\;(1\;-N)\tau^{*}$$

(11) $$N=(L_{1}\cdot {\;L}_{i})\;\times \;(s_{1}\cdot {\;s}_{i})$$

where L_1_ and *Li* denote the intersection lines of the grain boundary with the slip planes of the incoming and emitted dislocations, respectively, and *s*_1_ and *s*_i_ represent the corresponding slip directions. The grain boundary is estimated to possess a maximum obstacle stress $\tau^{*}$ of 1.1 GPa in body-centered cubic (BCC) metals [33,40]. Since Fe-3% Si non-oriented silicon steel has a BCC structure and the deformation mechanisms are identical to those in other BCC metals, this value is directly applicable to our model. For each incoming slip system, the obstacle stress *τ*_obs_ is taken as the smallest value among all permissible emitted slip systems. In the present study, 12 × {110}<111>, 12 × {112}<111> slip systems are considered for non-oriented silicon steel.

The evolution of slip system strength $g^{\alpha }$ describes the strain-hardening behavior:

(12) $${\dot{g}}^{\alpha }\;=\;\sum_{\beta }h_{\alpha \beta }{\dot{\gamma }}^{\beta }$$

(13) $$h_{\alpha \alpha }=h\left(\gamma \right)=h_{0}{\mathrm{sech}}^{2}\left|h_{0}\gamma |/|{(\tau }_{s}-\;\tau_{0})\right|$$

(14) $$h_{\alpha \beta }=\mathrm{qh}(\gamma )$$

The self- and latent-hardening contributions are represented by $h_{\alpha \alpha }$ and $h_{\alpha \beta }$ (*α* ≠ *β*) respectively. The associated material constants are given by $h_{0}$ = 60, $\tau_{0}$ = 161 MPa, $\tau_{s}$ = 1137 MPa and *q* = 1.4, with *γ* being the cumulative shear strain on all slip systems. The crystal plasticity model was implemented as a standalone Fortran program and compiled using the GFortran 12.2 compiler.

### 2.2. Experimental Method

Quasi-in situ EBSD analysis was employed to investigate the influence of neighboring grain orientations. A Fe-3.0 wt.% Si non-oriented silicon steel sheet with dimensions of 20 mm (RD) × 10 mm (TD) × 1 mm (ND) was prepared by mechanical polishing, followed by electropolishing in a solution of 96% ethanol and 4% perchloric acid at 15 V for 20 s to remove the surface deformation layer introduced by mechanical polishing. Prior to deformation, the initial orientations and the coordinates of a selected region on the ND–RD plane, measured relative to the sample edges, were recorded by EBSD. The sample was then cold-rolled with a 10% thickness reduction using a . After deformation, the sample was re-examined by EBSD without subsequent polishing. The same region was preliminarily located by converting the pre-recorded edge-referenced coordinates to account for the dimensional changes induced by rolling, and the exact correspondence of individual grains before and after deformation was further confirmed based on grain morphology. Grain orientations before and after deformation were characterized using a Crossbeam 550 scanning electron microscope (SEM, JEOL, Tokyo, Japan) equipped with an EBSD system. The step size was set to 3 μm for the initial state and 0.2 μm for the deformed state in order to capture the detailed orientation features within the grain-boundary regions. Orientation image maps were analyzed using AztecCrystal 3.1 (Oxford Instruments, Abingdon, UK).

## 3. Results

### 3.1. Orientation Rotation in Grain-Boundary Regions: Simulation and Experiment

**Figure 1:**
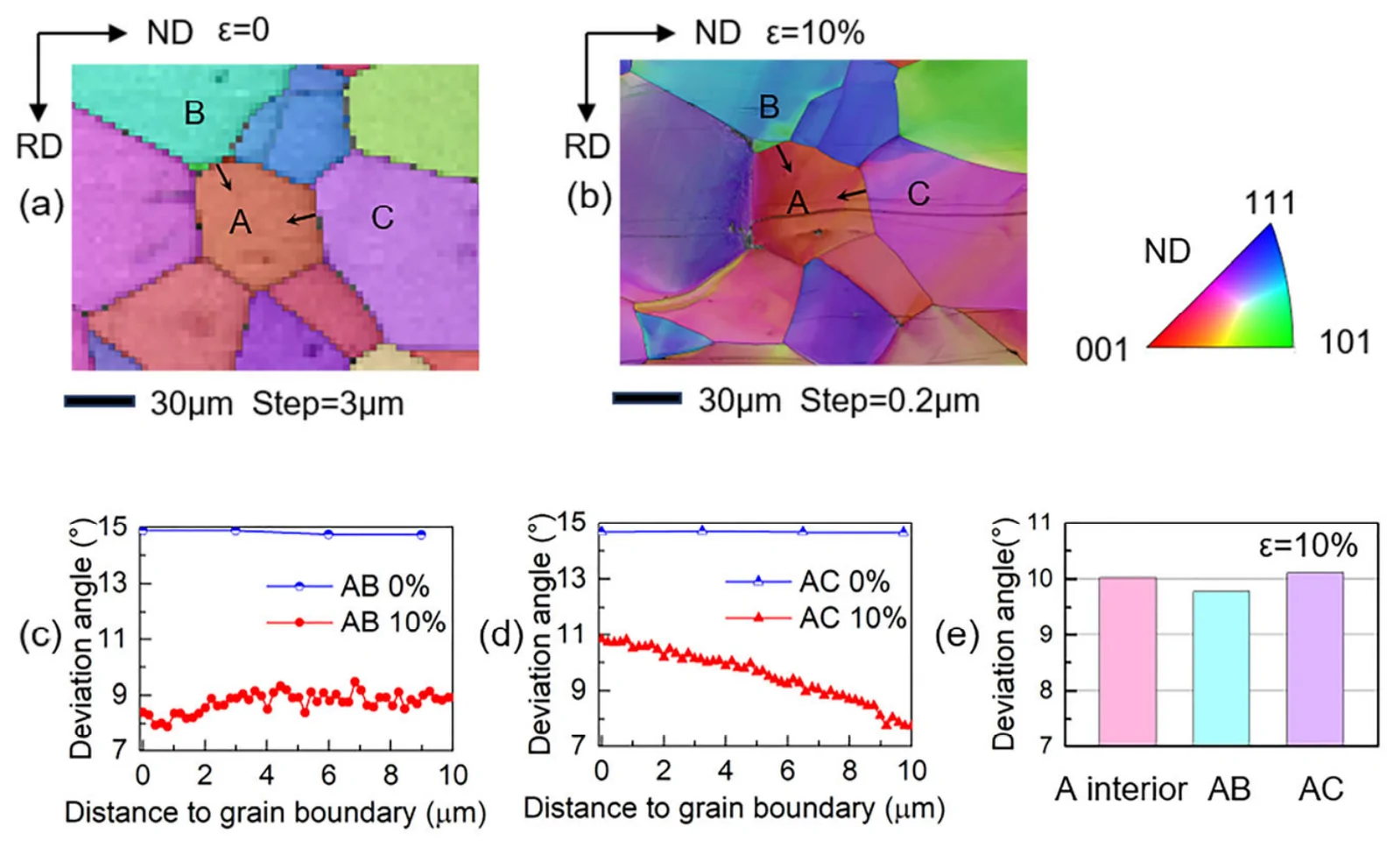
Experimental orientation image maps in the cold-rolled Fe-3.0 wt.% Si non-oriented silicon steel sheet obtained by EBSD (**a**) in the initial state and (**b**) at a 10% rolling reduction. Experimental deviation angle from the ideal λ orientation along lines perpendicular to the grain boundary between (**c**) grains A and B and (**d**) grains A and C. (**e**) Calculated deviation angle from the λ orientation in the grain interior and grain-boundary regions of grain A after deformation.

Quasi-in situ EBSD analysis was performed to track orientation rotation in the grain interior and grain-boundary regions. As shown in Figure 1, grain A (*φ*_1_ = 37°, Φ = 77°, *φ*_2_ = 5°) was adjacent to grains B (*φ*_1_ = 5°, Φ = 46°, *φ*_2_ = 22°) and C (*φ*_1_ = 34°, Φ = 71°, *φ*_2_ = 27°). Test points along a line perpendicular to the grain boundary represent the grain-boundary region and the grain interior, respectively. Prior to deformation, grain A exhibited a nearly uniform deviation angle of approximately 15° from the ideal λ orientation. After deformation, the deviation angle decreased noticeably both in the grain interior and in the grain-boundary regions, indicating that grain A rotated toward the ideal λ orientation. Nevertheless, the neighboring orientations exerted distinct influences on the grain-boundary regions. In the region adjacent to grain B, the deviation angle increased significantly with increasing distance from the boundary, suggesting that orientation B promoted the convergence of grain A toward λ. In contrast, in the grain-boundary region adjacent to grain C, the deviation angle was markedly larger than that in the grain interior, implying that orientation C hindered this convergence. The crystal plasticity simulation results in Figure 1e agree well with the experimental observations, thereby validating the accuracy of the computational model employed in this study. While the experimental validation is limited to a representative local cluster, its purpose is to verify the model’s core mechanisms, thereby providing confidence in the subsequent simulation-based statistical analysis across the broader orientation space.

### 3.2. Oriented Stability in Grain-Boundary Regions Affected by Neighboring Orientations

**Figure 2:**
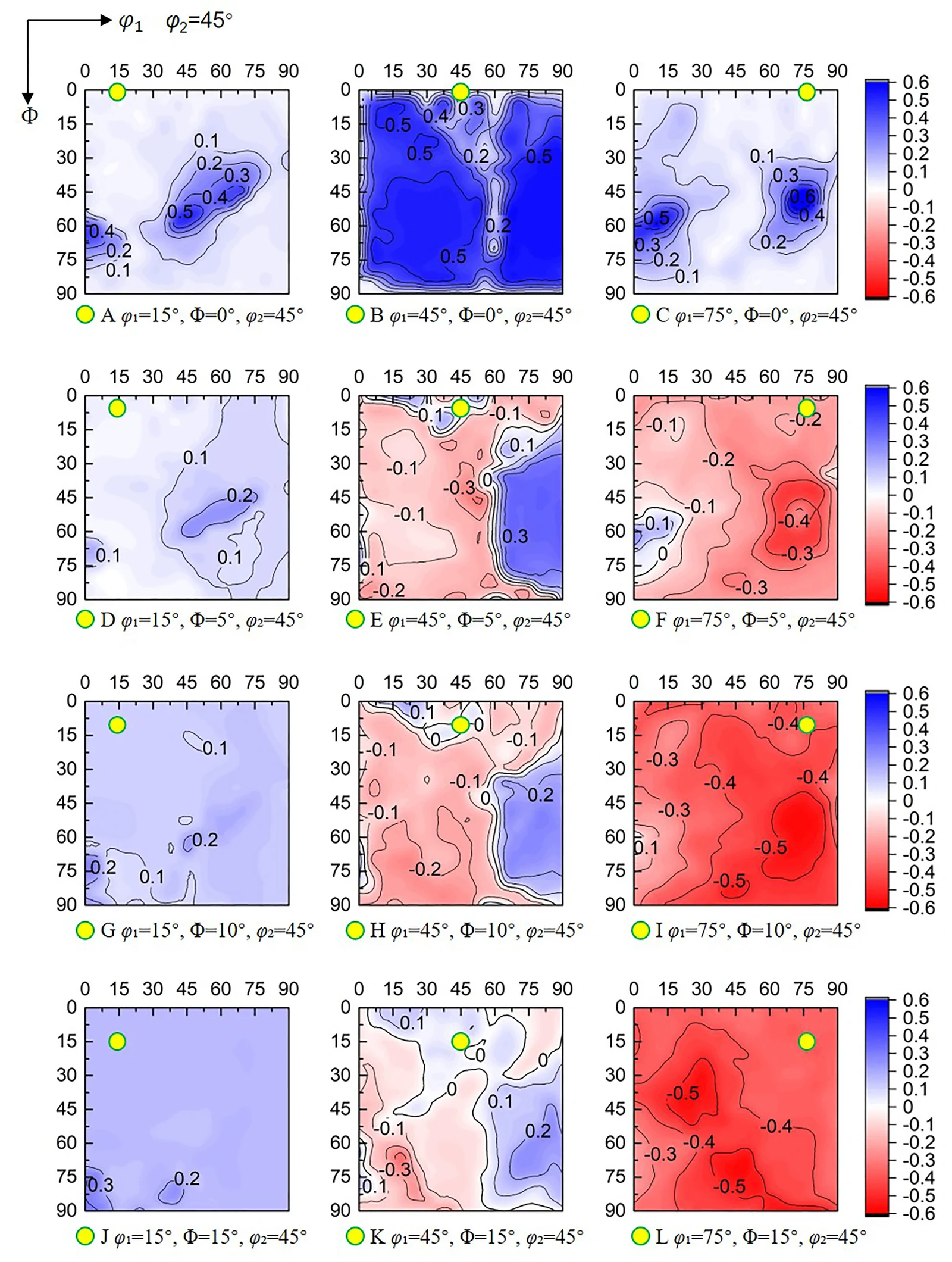
Oriented stability in grain-boundary regions of initial orientations A–L under the influence of neighboring orientations on the *φ*_2_ = 45° section. The yellow dot indicates the position of the initial orientation.

In non-oriented silicon steel, the λ deformation texture originates from the retention of initially scattered λ orientations during cold rolling. Accordingly, orientations deviating within 15° from the ideal λ fiber were selected as representative initial orientations in Figure 2. A (φ_1_ = 15°, Φ = 0°, φ_2_ = 45°), B (45°, 0°, 45°) and C (75°, 0°, 45°) are the ideal λ orientations. D (15°, 5°, 45°), E (45°, 5°, 45°) and F (75°, 5°, 45°) deviate from λ by 5°; G (15°, 10°, 45°), H (45°, 10°, 45°) and I (75°, 10°, 45°) deviate by 10°; J (15°, 15°, 45°), K (45°, 15°, 45°) and L (75°, 15°, 45°) deviate by 15°. Neighboring orientations were sampled at 5° intervals over the *φ*_2_ = 45° section in Euler space, covering all major texture components in BCC metals.

For the ideal λ initial orientations A, B and C, the oriented stability in the grain interior is approximately zero. Under the influence of neighboring orientations, the oriented stability in grain-boundary regions becomes positive. In particular, neighboring orientations near the γ fiber (*φ*_1_ = 0–90°, Φ = 55°, *φ*_2_ = 45°) cause a marked increase in oriented stability for orientations A and C. For initial orientations E, H and K, the grain interior exhibits a low negative oriented stability. In grain-boundary regions, the response to neighboring orientations is highly diverse: the oriented stability becomes strongly positive under the influence of neighboring orientations within (*φ*_1_ = 60–90°, Φ = 30–80°, *φ*_2_ = 45°), and highly negative under the influence of those within (*φ*_1_ = 0–45°, Φ = 30–80°, *φ*_2_ = 45°).

For initial orientations F, I and L, both the grain interior and grain-boundary regions exhibit negative oriented stability. Neighboring orientations near (φ_1_ = 60°, Φ = 55°, φ_2_ = 45°) substantially increase the magnitude of this negative oriented stability for orientations F and I. In contrast, for initial orientations D, G and J, the oriented stability in both the grain interior and grain-boundary regions is low and positive, and it is insensitive to neighboring orientations.

### 3.3. Deformation Orientations in Grain-Boundary Regions Affected by Neighboring Orientations

**Figure 3:**
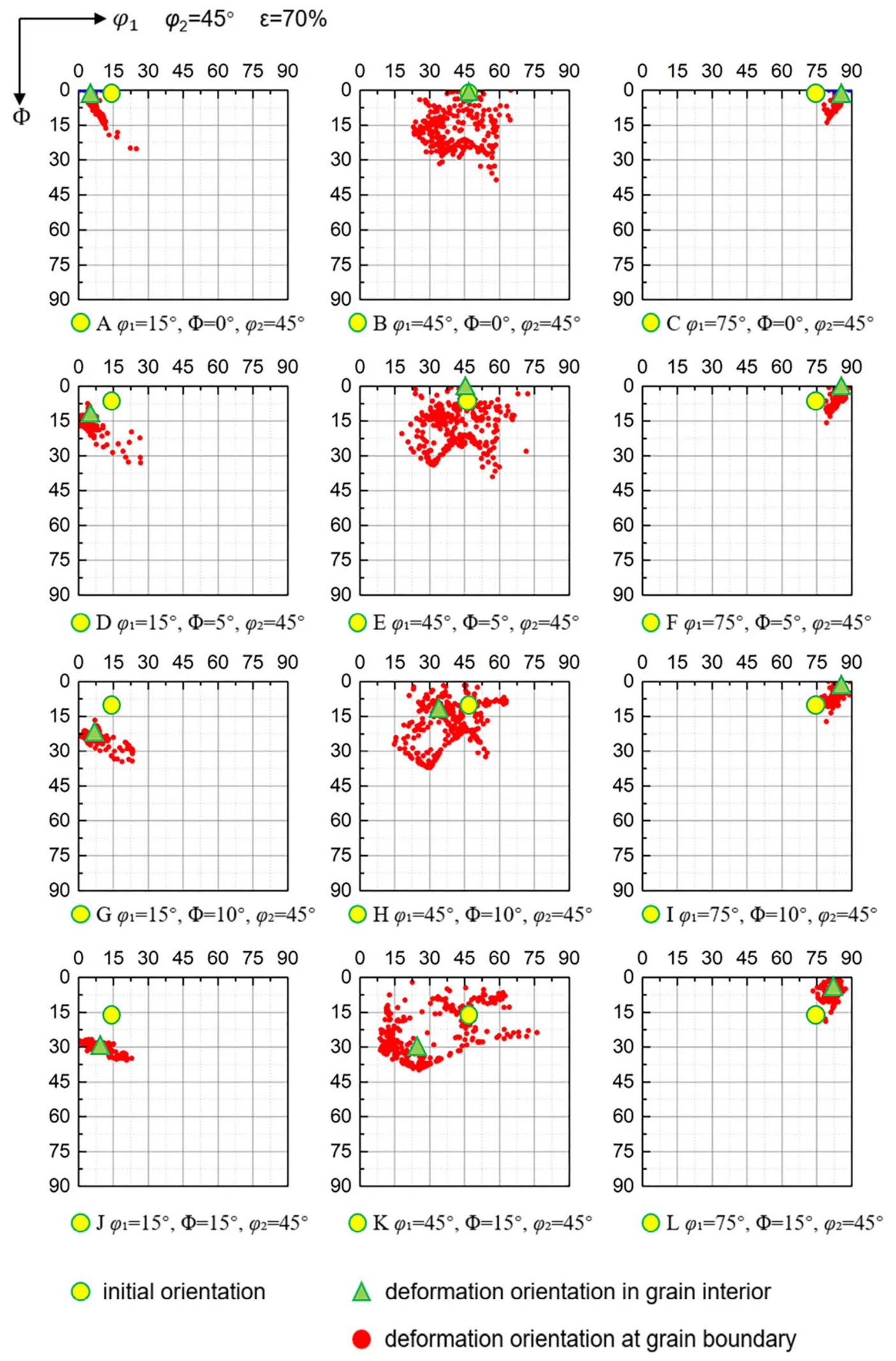
Deformation orientations in grain-boundary regions of initial orientations A–L under the influence of neighboring orientations on a *φ*_2_ = 45° section after 70% cold rolling. The yellow dot, green triangle, and red dot represent the positions of the initial orientation, the deformation orientation in the grain interior, and the deformation orientation in grain-boundary regions, respectively.

Neighboring orientations influence the rotation path in grain-boundary regions and consequently alter the deformation orientations after plastic deformation. For the initial and neighboring orientations selected in Figure 2, Figure 3 presents the deformation orientations both in grain interiors and in grain-boundary regions after cold rolling to a 70% reduction. The experimental rolling reduction is limited to 10% in Figure 2 to ensure reliable EBSD pattern indexing and clear identification of grain-boundary regions, whereas the simulations in this section are extended to 70% to predict the deformation texture at the industrial rolling reduction. The model, having been validated at 10% strain, is then employed for forward prediction at the higher reduction. Since the ideal λ orientation lies on the Φ = 0 axis, the angular deviation of a deformed orientation from the Φ = 0 axis equals its deviation from the target λ orientation.

In Figure 3, orientations B, E, H and K exhibit high sensitivity to neighboring orientations. In grain interiors, these orientations approximately retain their initial positions in Euler space after rolling, whereas their deformation orientations deviate markedly from the starting positions when influenced by neighboring orientations. As shown in Figure 2, the oriented stability of orientation B becomes positive under the influence of neighboring orientations; correspondingly, the deformation orientations of orientation B display a clear deviation from the ideal λ orientation. For orientations E, H, and K, the oriented stability can become highly positive or negative depending on the neighboring orientation, causing the angular deviation from the target λ orientation after deformation to either increase or decrease. In contrast, orientations A, D, G, J and C, F, I, L are insensitive to neighboring orientations, exhibiting nearly identical deformation orientations in both grain interiors and grain-boundary regions.

## 4. Discussion

### 4.1. Dependence of Oriented Stability in Grain-Boundary Regions on Initial Orientation

**Figure 4:**
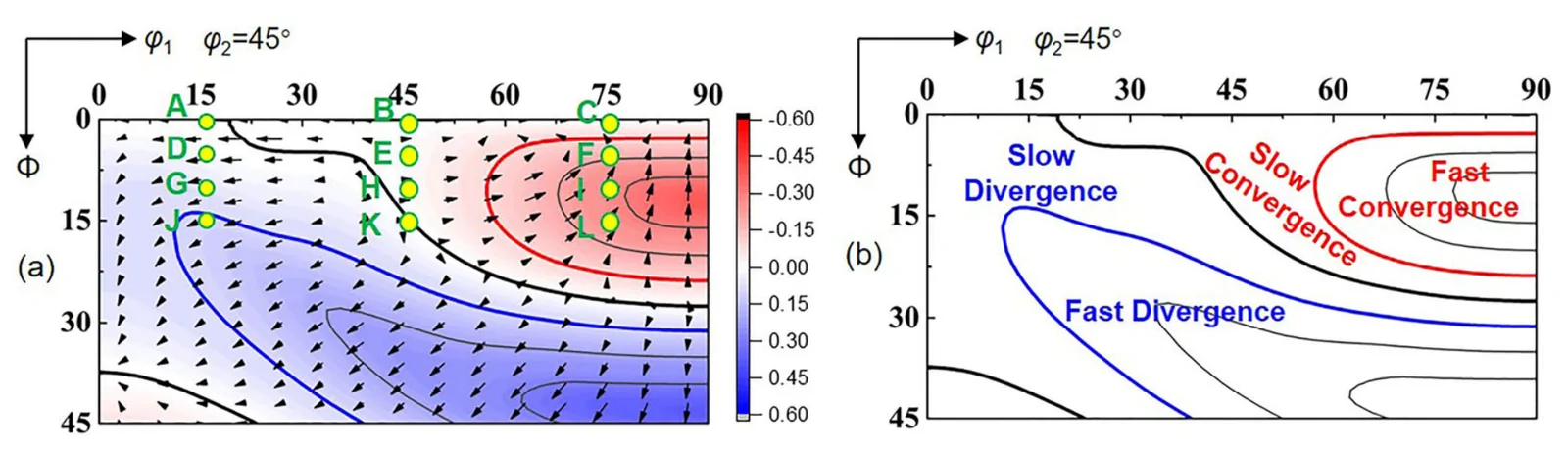
(**a**) Oriented stability distribution of scattered {001} ⟨uv0⟩ orientations in the grain interior. (**b**) Schematic diagram of different orientation zones. Arrows indicate the magnitude and direction of the rotation vector. Panels A–L represent the initial orientations.

The oriented stability in grain-boundary regions is highly sensitive to the initial orientation, a sensitivity that is intrinsically related to the oriented stability in the grain interior. Figure 4a presents the distribution of oriented stability for scattered λ orientations in the grain interior, as established in our earlier work [19]. Based on the magnitude of oriented stability, the initial orientations can be classified into four zones: fast divergence, slow divergence, slow convergence, and fast convergence. The boundaries of these zones are indicated by dark red, black, and blue lines, while the black arrows denote the rotation velocity field of the initial orientations. It can be observed that the rotation paths of initial orientations within the divergence zones remain consistently inside those zones, whereas orientations in the fast convergence zone always rotate within the convergence zone. However, orientations near the critical boundary (black line) that separates the convergence and divergence zones exhibit diverse rotation trends.

Initial orientations B, E, H, and K lie in the vicinity of this critical boundary. In this region, even slight perturbations of the rotation paths caused by neighboring grains can lead to distinctly different oriented stability in the grain-boundary regions, rendering it highly sensitive to the neighboring orientation. In contrast, initial orientations A, D, G, and J are located within the divergence zone; the changes in rotation paths induced by neighboring grains are insufficient to significantly alter their oriented stability, so that their oriented stability in the grain-boundary regions is insensitive to neighboring orientations. Similarly, initial orientations C, F, I, and L lie in the fast convergence zone, far from the critical boundary, and are also insensitive to neighboring orientations; neighboring orientations can only alter the magnitude, but not the sign, of their oriented stability.

### 4.2. Contribution of Neighboring Orientations to Oriented Stability in Polycrystals

For a given initial orientation, neighboring orientations exert varying influences on the oriented stability in grain-boundary regions. In textured polycrystals, the contribution of each neighboring texture component is directly related to its volume fraction. To quantify the statistical effect of neighboring texture component *j* on the oriented stability of initial orientation *i*, the contributed oriented stability, ${\dot{\theta }}_{\mathrm{ij}}^{C}$, is defined as

(15) $${\dot{\theta }}_{\mathrm{ij}}^{C}={{\;v}_{j}\dot{\theta }}_{\mathrm{ij}}$$

where *v_j_* is the volume fraction of component *j*. Thus, the contributed oriented stability depends on the texture state: a higher volume fraction of a given neighboring component yields a greater contributed oriented stability.

**Figure 5:**
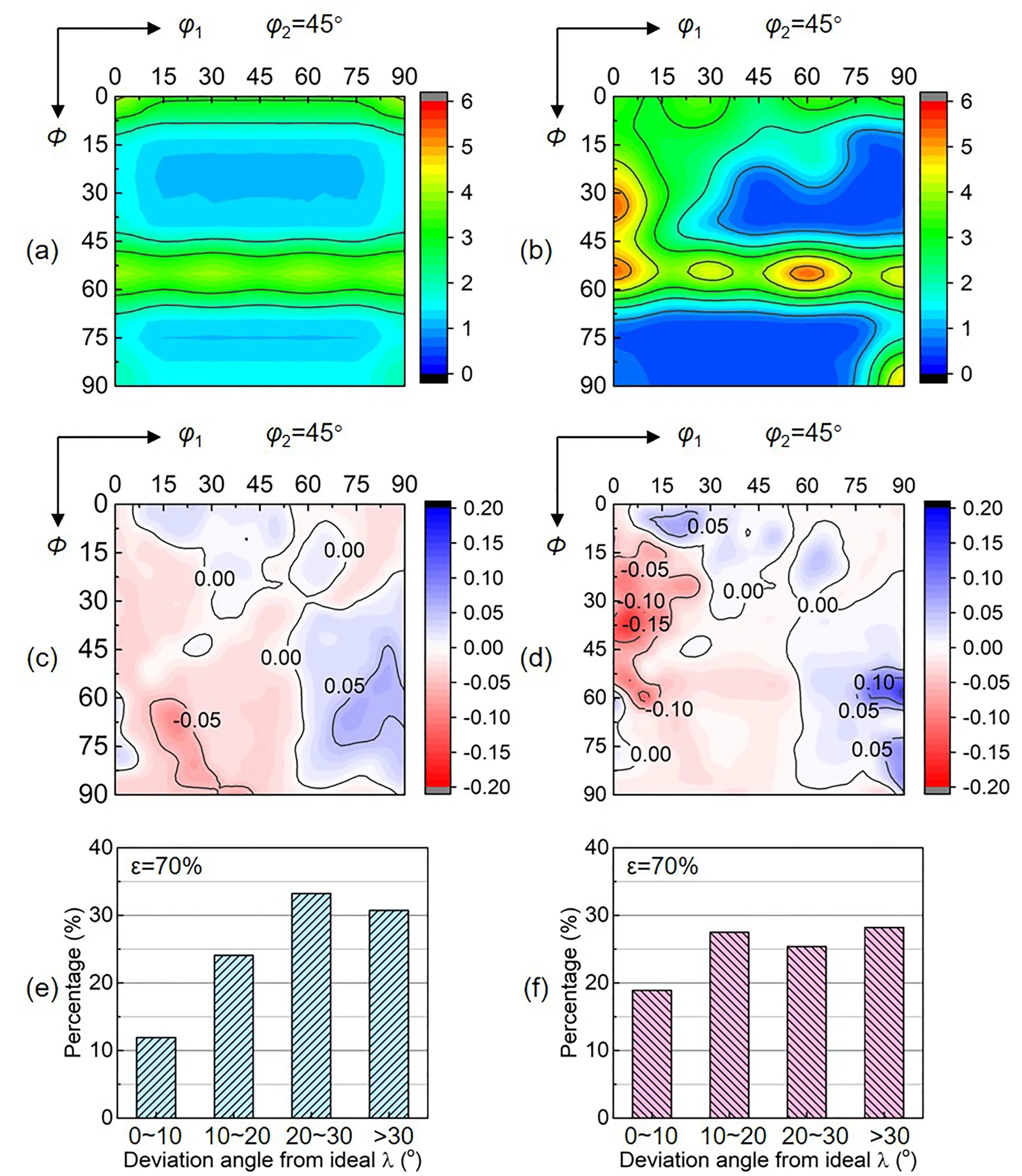
Orientation distribution function on a constant *φ*_2_ = 45° section for initial (**a**) near-random texture and (**b**) rolling texture; contributed oriented stability distribution for initial (**c**) random texture and (**d**) rolling texture; and simulated deviation angle distribution from the ideal λ orientation for initial (**e**) random texture and (**f**) rolling texture. The initial orientation is (*φ*_1_ = 45°, Φ = 15°, *φ*_2_ = 45°).

Figure 5 shows the contributed oriented stability of various neighboring texture components on the constant *φ*_2_ = 45° section, taking the initial orientation K (*φ*_1_ = 45°, *Φ* = 15°, *φ*_2_ = 45°), which is sensitive to the neighboring orientation, as an example. Two initial texture states are examined. Figure 5a presents a near-random texture, with equal volume fractions assigned to all discrete orientation points in Euler space. However, when both crystal symmetry and sample symmetry are considered in the calculation of the orientation distribution function (ODF), densities accumulate along the γ-fiber. In contrast, Figure 5b exhibits a typical rolling texture. Compared with the initial near-random texture, the rolling texture has a higher volume fraction of neighboring orientations near (*φ*_1_ = 0°, *Φ* = 35°, *φ*_2_ = 45°), which therefore contribute more negative oriented stability. Consequently, the initial orientation K exhibits different fractions of the deviation angle under the two texture states. In the rolling texture, the fractions of this orientation with deviation angles within 10° and within 20° increase significantly, owing to the influence of the neighboring component (*φ*_1_ = 0°, *Φ* = 35°, *φ*_2_ = 45°). While grain-boundary character, spatial connectivity, and neighbor-pair correlations may introduce quantitative variations, they are not expected to alter the qualitative ranking of oriented stability established by our volume-fraction-based parameter. Therefore, the distribution of contributed oriented stability provides a basis for designing neighboring orientations to efficiently control deformation textures in grain-boundary regions.

## 5. Conclusions

(1) The difference in oriented stability between grain interiors and grain-boundary regions depends on the initial orientation. Initial orientations that lie near the critical boundary separating convergence and divergence zones exhibit extreme susceptibility to neighboring orientations, which can even reverse the rotation direction relative to the target λ orientation. In contrast, for initial orientations far from this critical boundary, the influence of neighboring orientations is substantially weaker and only modifies the magnitude of the oriented stability.

(2) The influence of neighboring orientations on the oriented stability in grain-boundary regions is directional and selective. For the sensitive initial orientations near the critical boundary, the response to neighboring orientations is highly diverse: neighboring orientations located within *φ*_1_ = 0–45°, Φ = 30–80°, *φ*_2_ = 45° favor convergence of the initial orientation toward the ideal λ fiber, whereas those within *φ*_1_ = 60–90°, Φ = 30–80°, *φ*_2_ = 45° promote divergence away from λ. This spatial criterion provides a precise basis for distinguishing beneficial from detrimental neighboring texture components.

(3) Contributed oriented stability integrates both the intrinsic oriented stability of the initial orientation and the volume fraction of each neighboring component to quantify the statistical effect of different neighboring texture components in polycrystals, thereby providing an effective pathway for texture optimization in non-oriented silicon steel. First, the contributed oriented stability maps can guide the selection of initial texture components that inherently tend to rotate toward the favorable λ fiber. Moreover, this parameter enables the quantitative design of the local texture environment to steer the grain-boundary regions toward the favorable λ texture during rolling.

## Data Availability

The original contributions presented in this study are included in the article. Further inquiries can be directed to the corresponding authors.

## Abbreviations

The following abbreviations are used in this manuscript:

EBSD	Electron backscatter diffraction
ND	Normal direction
GNDs	Geometrically necessary dislocations
KAM	Kernel average misorientation
GROD	Grain reference orientation deviation
GAS	Gradient average severity
BET	Boundary effective thickness
BCC	Body-centered cubic
